# Three new species of the carnivorous snail genus *Perrottetia* Kobelt, 1905 from Thailand (Pulmonata, Streptaxidae)

**DOI:** 10.3897/zookeys.287.4572

**Published:** 2013-04-11

**Authors:** Thanit Siriboon, Chirasak Sutcharit, Fred Naggs, Somsak Panha

**Affiliations:** 1 Biological Science Program, Animal Systematics Research Unit, Department of Biology, Faculty of Science, Chulalongkorn University, Bangkok 10330, Thailand; 2 Department of Life Sciences, The Natural History Museum, Cromwell Road, London SW7 5BD, United Kingdom

**Keywords:** Systematics, land snails, taxonomy, genitalia, predator

## Abstract

Three new species of the streptaxid snail genus *Perrottetia* are described from north and northeastern Thailand, *Perrottetia aquilonaria* **sp. n.**, *Perrottetia dermapyrrhosa* **sp. n.** and *Perrottetia phuphamanensis* **sp. n.** Each species is endemic to a single or a few limestone mountain ranges. The species are characterized by the morphology of their genital organs, as well as by shell characters. *Perrottetia aquilonaria* **sp. n.** has a club shaped distal penis and large penial hooks are present and penial papillae cover almost the entire penial hook portion; adjacent areas possess low reticulated folds. *Perrottetia dermapyrrhosa* **sp. n.** has a long genital atrium and the penial sheath is about two-thirds of the penis length. Penial hooks are long, scattered and sunken into deep ovate hollows; vaginal hooks are present. *Perrottetia phuphamanensis* sp. n. has a rounded and protruded shell periphery. The aperture is subcircular, peristome is thick and the second parietal lamella is adjacent to the first parietal lamella; a basal lamella is the smaller than in the other Thai species.

## Introduction

Terrestrial gastropods are primarily herbivores and only a few groups are carnivorous. Carnivorous snails usually feed on other snail species or on weak individuals of the same species; some feed on insect larvae or earthworms (Gray 1860, Blanford and Godwin-Austen 1908, Benthem Jutting 1954, Berry 1963). Most diverse among tropical Asian carnivorous snails are members of the speciose family Streptaxidae Gray, 1860. Streptaxids can generally be recognized by their eccentric or cylindrical shells, while the animals have a bright yellow to red or orange body with external hook-like structures on the everted penis (Verdcourt 2000, Schileyko 2000, Rowson et al. 2009). The family is widely distributed across the tropical and subtropical areas of South America, Africa and Asia (Bruggen 1967, Schileyko 2000, Sutcharit et al2010). Currently, the Streptaxidae are divided into 6 subfamilies comprising some 60 genera and about a thousand nominal species (Zilch 1960, Richardson 1988, Schileyko 2000). In the recent decades, most of the taxonomic and systematic research on streptaxids has been performed on sub-Saharan African taxa, where the species diversity reaches its maximum (Rowson et al. 2010). Only a few publications focus on South American or Asian groups (Barbosa et al. 2008, references therein; Clements 2006). Recently, the deep phylogenetic structure of the Streptaxoidea has been revealed, resulting amongst others in the recognition of a Southeast Asian lineage as a distinct family, the Diapheridae Panha & Naggs, 2010 (Sutcharit et al. 2010, Rowson et al. 2010).

With 13 genera and about 130 nominal species, the second most diverse streptaxid fauna can be found in Southeast Asia (Bruggen 1967, 1972, Richardson 1988, Schileyko 2000). *Perrottetia* Kobelt, 1905 is a poorly known genus with 27 nominal species. Most species of *Perrottetia* live in India, but there are additional species of this genus known from Sri Lanka, Laos, Vietnam and Southern China (Gude 1903, Kobelt 1906, Blanford and Godwin-Austen 1908, Zilch 1961, Richardson 1988, Schileyko 2000, 2011). To date, all nominal species of *Perrottetia* were described over a century ago and were often based on brief descriptions with poorly detailed figures.

The most prominent characters of *Perrottetia* are the sub-oblique heliciform shell, often with whorls coiling around an oblique axis. The last whorls do not descend below the preceding whorl, and short longitudinal furrows are present behind the apertural lip. Internally, the aperture possesses two parietal lamellae (Kobelt 1906, Zilch 1960, Richardson 1988, Schileyko 2000). Apart from a description of the anatomy of the Indian species *Perrottetia gudei* (Fulton, 1915) by Schileyko (2000), all other taxonomic studies on *Perrottetia* have solely been based on shell characters. The characters of the genitalia, in particular those proximal to the genital orifice, have proved as useful tools for species recognition, and often can serve for the differentiation on the generic level in many streptaxids groups (Stoliczka 1871, Berry 1963, Sutcharit et al. 2010). Subsequently, these characters were also examined in the current investigation.

## Material and methods

**Figure 1.**
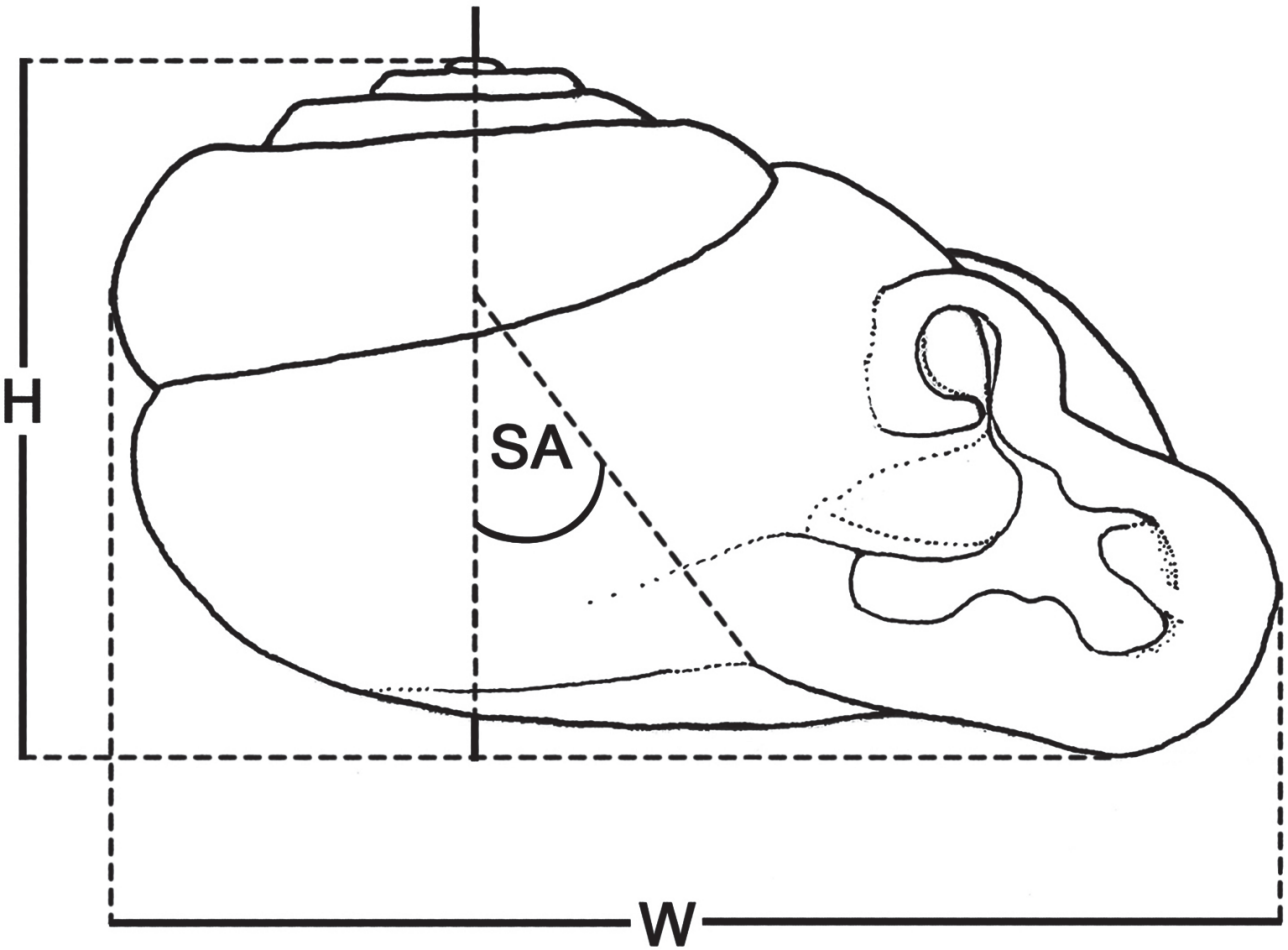
Schematic diagram illustrating methods for measuring specimens: **H** shell height **SA** shell angle **W** shell width.

Our faunistic surveys throughout Thailand from 2008–2012 yielded rich collections of both, shells and live streptaxids, from north and northeast Thailand. Based on their distinctive shell characters, three new *Perrottetia* species are recognized. In addition to shell characters we examined the genitalia and radulae. Identifications were provisionally based on Tryon (1885), Blanford (1899), Kobelt (1906) and Blanford and Godwin-Austen (1908) prior to comparison with relevant type specimens. Living snails were photographed, refrigerated in -20 °C for approximately 6 hours prior to preservation in 70% ethanol for anatomical study. Shell height (H), shell width (W) and shell angle (SA) were measured as shown in Figure 1. Shells were digitally imaged using Cell’D Imaging Software. The genitalia of 5–10 specimens of each species were dissected and examined under a stereo-microscope. Representative examples of dissected specimens were drawn using a camera lucida. The buccal mass was removed, and the radulae were soaked in 10% sodium hydroxide, cleaned in distilled water, examined and photographed under SEM (JEOL, JSM-5410 LV). For examination of penial and vaginal hooks under SEM (PHILIPS, XL30), tissues were critical point dried from absolute ethanol.

The nomenclature of the shell apertural dentition follows that of Pilsbry (1916). In the descriptions of features of the genital organs, ‘proximal’ relates to the genital orifice, and ‘distal’ to the region furthest away from the genital orifice. The new anatomical terms ‘vaginal hook’ and ‘atrial pore’ are introduced. Others characters are as defined by Stoliczka (1871), Berry (1963), Verdcourt (2000), Herbert (2002) and Sutcharit et al. (2010): ag, albumen gland; at, atrium; fo, free oviduct; gd, gametolytic duct; gs, gametolytic sac; hd, hermaphroditic duct; ov, oviduct; p, penis; pr, penial retractor muscle; ps, penial sheath; psr, penial sheath retractor muscle; sv, seminal vesicle; ta, talon; v, vagina; vd, vas deferens.

Material examined in this study is deposited in the following institutions: CUMZ, Chulalongkorn University Museum of Zoology, Bangkok; NHMUK, The Natural History Museum, London; SMF, Forschungsinstitut und Naturmuseum Senckenberg, Frankfurt am Main.

All descriptions of the new species are here attribute to the first and the fourth author, Siriboon and Panha, respectively.

## Systematics

### Family Streptaxidae Gray, 1860

#### Genus Perrottetia

Kobelt, 1905

http://species-id.net/wiki/Perrottetia

Odontartemon (Perrottetia) Kobelt, 1905[1906]: 91, 108. Thiele 1931: 730. Forcart 1946: 215.
Oophana (Perrottetia) – Benthem Jutting 1954: 95.
Perrottetia – Zilch 1960: 562, 563. Schileyko 2000: 777, 778.

##### Type species.

*Helix peroteti* Petit, 1841 by subsequent designation of Forcart (1946: 215).

##### Description.

Theshell is oblique-heliciform, usually thin and opaque. Its surface is smooth and glossy but fine transverse ridges may be present. The embryonic shell is smooth. The 5–7 whorls increase regularly. The shell periphery is usually rounded and the last whorl does not descend below the preceding whorl but is parallel to the preceding suture. The outer wall of the last whorl generally possesses two short longitudinal furrows that correspond with internal apertural lamellae. The umbilicus is narrow and deep. The semi-ovate aperture has an expanded peristome with a reflexed lip. The apertural dentition consists of two parietal lamellae; palatal, basal and columellar lamellae are usually present; upper palatal and supracolumellar lamellae may also be present.

Living animals possess a yellowish to reddish reticulated skin. The brown digestive gland and the black kidney are visible through the transparent shell. The upper tentacles are longer than the lower pair with a black eye-spot on the tip of the fully extended tentacle; bright red or yellow retractor muscles show though the transparent skin. The foot is narrow, undivided, the tail short.

Genitalia with a long, slender penis; penial sheath short, about half of penis length; internal wall of introverted penis with black to brown penial hooks; vas deferens passes through a short section of penial sheath before connecting distally to penis; vagina and free oviduct short to long, vaginal hooks may be present; gametolytic duct and sac may not extend as far as albumin gland; seminal vesicle present with about the same length from vesicle to talon.

##### Remarks.

*Perrottetia* consists of 27 nominal species distributed across Southern Asia, southern China to northern Vietnam. So far, it was not recorded from Thailand (Kobelt 1906, Yen 1939, Richardson 1988, Panha 1996, Schileyko 2000, Hemmen and Hemmen 2001). “*Streptaxis siamensis* Pfeiffer, 1862” was provisionally placed in *Perrottetia* by Kobelt (1906), but the presence of a single large parietal lamella, and the absence of short longitudinal furrows on the outer wall of the last whorl clearly differentiate this species from *Perrottetia*. Examination of the genitalia of topotype specimens show that this species has a very long penis, with a large proximal gametolytic duct, and short penial hooks that are located on an undulated penial papillae. This indicates that “*Streptaxis siamensis* Pfeiffer, 1862” should be placed in *Oophana* Ancey, 1884 (further details of this will be published elsewhere).

The first description of the genital system of a member of the subfamily Streptaxinae Gray, 1860 was published by Gerlach and van Bruggen (1999). Schileyko (2000) included 21 genera within the Streptaxinae. Two of them, *Streptartemon* Kobelt, 1905, from South America and *Seychellaxis* Schileyko, 2000, from the Seychelles, share the oblique-heliciform shell, and their penis sheath closely resembles that of *Perrottetia*. However, these may be plesiomorphic character states, and no phylogenetic affinity can be inferred.

#### Perrottetia dermapyrrhosa

Siriboon & Panha sp. n.

urn:lsid:zoobank.org:act:029F7FDD-9A8A-4B36-A782-815BFA0D32EB

http://species-id.net/wiki/Perrottetia_dermapyrrhosa

**Figure 2.**
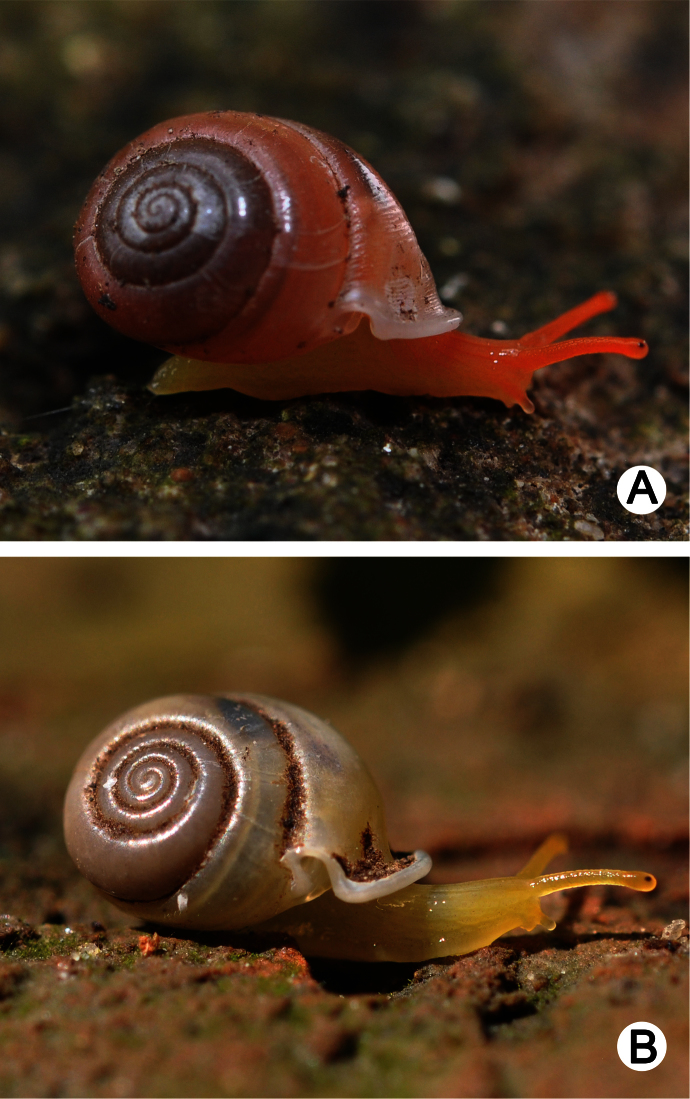
Living snails of **A** *Perrottetia dermapyrrhosa* sp. n.(paratype CUMZ 5002) from the type locality (shell width about 7 mm), and **B** *Perrottetia aquilonaria* sp. n. (paratype CUMZ 5004) from the type locality (shell width about 6 mm).

Figs 2A 3A–C 4A–C 5A–G 6A Table 1

##### Type material:

**Figure 3.**
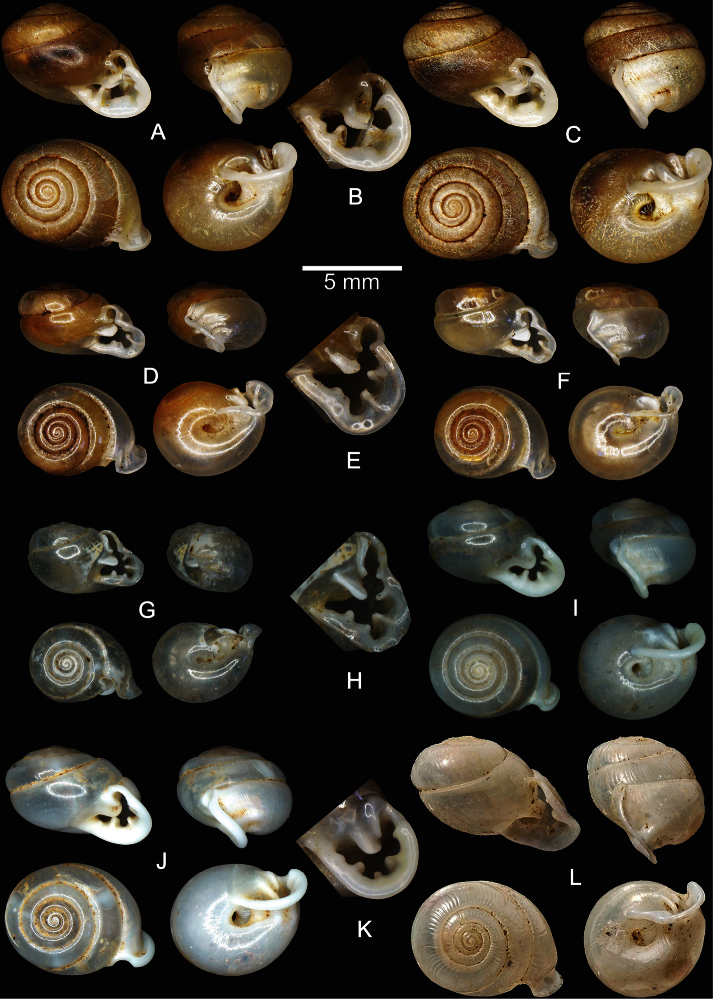
Shells of *Perrottetia* spp. **A–C** *Perrottetia dermapyrrhosa* sp. n. **A** holotype CUMZ 5001 **B** apertural dentition of the holotype CUMZ 5001, and **C** paratype CUMZ 5002 **D–H** *Perrottetia aquilonaria* sp. n. **D** holotype CUMZ 5003 **E** apertural dentition of the holotype CUMZ 5003 **F** paratype CUMZ 5004 **G** specimen from Tam Chiangdao, Chiangmai, CUMZ 5008 and **H** apertural dentition of the specimen from Tam Chiangdao, Chiangmai CUMZ 5008 **I–K** *Perrottetia phuphamanensis* sp. n. **I** holotype CUMZ 5011 **J** paratype CUMZ 5012, and **K** apertural dentition of the holotype CUMZ 5011 **L** *Perrottetia gudei* Fulton, 1915, syntype NHMUK 1919.12.31.51.

Holotype CUMZ 5001 (Fig. 3A). Measurement: shell height 6.1 mm, shell width 7.7 mm, and with 6 whorls. Paratypes NHMUK 20130062 (2 shells), SMF 341486 (1 shell), CUMZ 5002 (2 shells).

##### Type locality.

Wat Tam Namsrithong, Nong Kungsi, Kalasin, Thailand, 16°48'18.0"N, 103°16'42.5"E.

##### Diagnosis.

*Perrottetia mabillei* (Bavay and Dautzenberg, 1903) can be distinguished from *Perrottetia dermapyrrhosa* sp. n. by its lower spire with a distinct suture. The left periphery of the penultimate whorl is shouldered and does not extend beyond the diameter of the last whorl. The aperture is triangular and a supracolumellar lamella is absent. In comparison, *Perrottetia peroteti* (Petit, 1841) possesses a lower spire with a distinct suture, fine transverse ridges are present and a smaller basal lamella, while upper palatal and supracolumellar lamellae are absent. *Perrottetia gudei* (Fulton, 1915) (syntype Fig. 3L) differs from *Perrottetia dermapyrrhosa* sp. n. in its lower spire, the second parietal lamella being smaller and shorter than the first lamella and an upper palatal lamella that is usually present (Petit 1841, Bavay and Dautzenberg 1903, Kobelt 1906, Fulton 1915). In *Perrottetia dermapyrrhosa* sp. n. the genital atrium is long, the penial sheath reaches about two-thirds of the penial length and the gametolytic duct and sac do not extend as far as the albumin gland. The penial hooks are more scattered and, in the introverted penis, are housed in deep ovate depressions; vaginal hooks are present. In comparison, *Perrottetia gudei* possesses a short genital atrium and penial sheath, and the gametolytic duct and sac extend as far as the albumin gland; the penial hooks are denser than in *Perrottetia dermapyrrhosa* sp. n., and each hook is situated on a small papilla (Schileyko 2000, fig. 1015D).

*Perrottetia dermapyrrhosa* sp. n. differs from *Perrottetia aquilonaria* sp. n. in its larger shell, which is less deviated from the vertical axis. A sinulus sensu Schileyko (2000) is absent; the first and second parietal lamellae are connected, and a bifid columellar and supracolumellar lamellae are absent. In comparison, *Perrottetia dermaphyrrhosa* sp. n. possesses a long atrium and vagina, and a penial sheath with a club shaped distal penis. The length of vas deferens that enters the penis distally is longer. The penial papillae are located in hollows, the penial hooks are much more scattered, and vaginal hooks are present.

##### Description.

Shell oblique-heliciform, white and translucent; whorls 6, spire conical, suture distinct; shell surface glossy, with transverse ridges that diminish below the periphery; embryonic shell large, consisting of about 2 whorls with smooth surface, following whorls regularly expanding; shell periphery rounded, last whorl axially deflected; two deep and short longitudinal furrows present; umbilicus narrow (Fig. 3A); aperture subcircular, peristome discontinuous, thickened and expanded; apertural dentition with a large transverse first parietal lamella, with second parietal lamella adjoined at right angles; one upper palatal lamella, one small palatal lamella, one large basal lamella, one long subcolumellar lamella, one large strong columellar lamella and one small supracolumellar lamella (Fig. 3B).

**Radula:** Teeth arranged in anteriorly V-shaped rows, each row contains 29–31 teeth with formula (14-15)-1-(14-15); central tooth very small and triangular with a pointed cusp; lateral and marginal teeth undifferentiated, unicuspid and lanceolate; lateral teeth gradually reducing in length and size; outer teeth much smaller and shorter than inner teeth (Fig. 6A).

**Figure 4.**
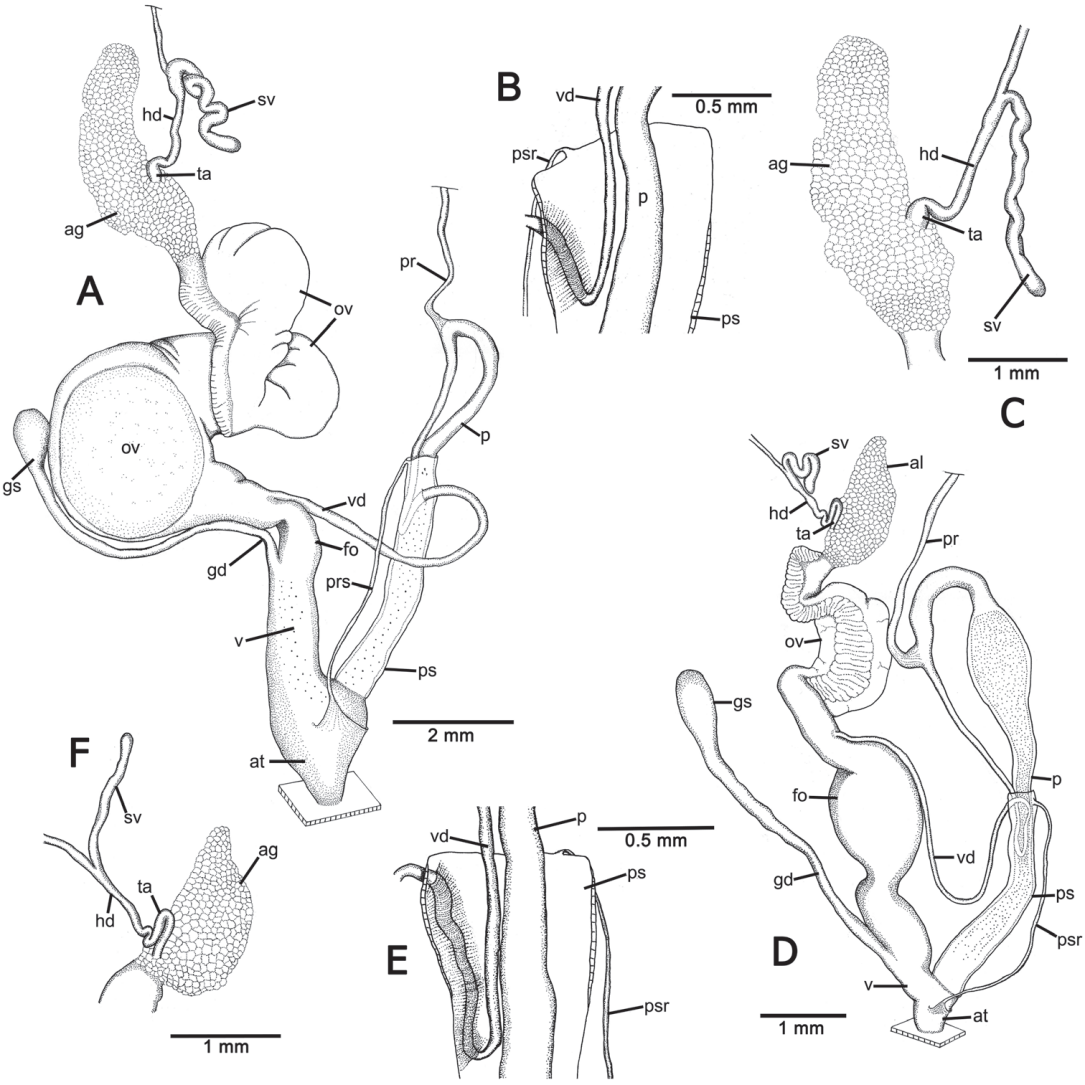
Genitalia of *Perrottetia* spp. **A–C** *Perrottetia dermapyrrhosa* sp. n. (paratype CUMZ 5002) **A** reproductive system **B** insertion of vas deferens into penial sheath, and **C** details of hermaphroditic duct and seminal vesicle **D–F** *Perrottetia aquilonaria* sp. n. (paratype CUMZ 5004), **D** reproductive system **E** insertion of vas deferens into penis sheath, and **F** details of hermaphroditic duct and seminal vesicle. Abbreviations: ag, albumen gland; at, atrium; fo, free oviduct; gd, gametolytic duct; gs, gametolytic sac; hd, hermaphroditic duct; ov, oviduct; p, penis; pr, penial retractor muscle; ps, penial sheath; psr, penial sheath retractor muscle; sv, seminal vesicle; ta, talon; v, vagina; vd, vas deferens.

**Figure 5.**
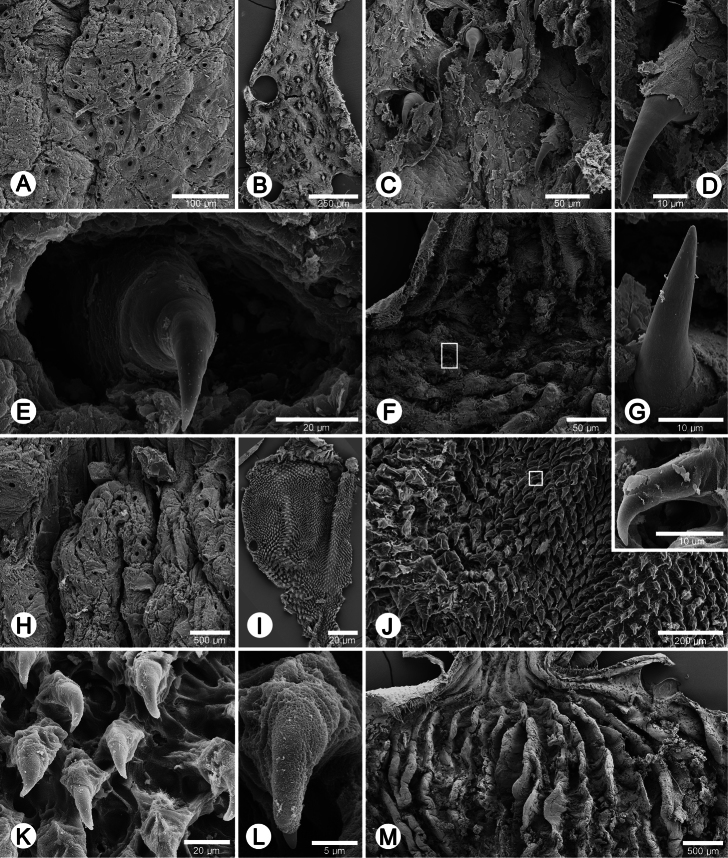
Internal sculpture of genitalia of *Perrottetia* spp. **A–G** *Perrottetia dermapyrrhosa* sp. n. (paratype CUMZ 5002) **A** details of atrial pore on the atrium surface **B** low magnification shows arrangement of penial hooks **C** high magnification of penial hooks **D** lateral view of penial hook **E** top view of penial hook situate inside hollow **F** arrangement of vaginal fold with hook in white square, and **G** lateral view of vaginal hook (from white square in **F**) **H–M**. *Perrottetia aquilonaria* sp. n. (paratype CUMZ 5004) **H** details of atrial pore on the atrium surface **I** low magnification shows dense arrangement of penial hooks **J** high magnification of penial hooks with (inset) lateral view of penial hook **K** arrangement of penial hooks **L** top view of penial hook, and **M** arrangement of vaginal folds without vaginal hook.

**Genital organs:** Atrium (at) long and slender; proximal penis (p) long, slender and with solid muscular penis sheath extending distally beyond penis sheath as a narrow tube; penial sheath (ps) reaching about two-thirds of total penis length, penial sheath retractor muscle very thin (psr), originating at atrium and inserting distally on penial sheath (Fig. 4A); vas deferens (vd) passes through about one-sixth of penial sheath length before entering into penis distally (Fig. 4B); penial retractor muscle (pr) thin and very long, inserting at penis and vas deferens junction; internal wall of atrium generally smooth with numerous pores (Fig. 5A); penial wall with scattered and pale brown penial hooks, about 3 hooks/200 µm^2^ (Fig. 5C), and hooks located on conical papillae surrounded by deep ovate hollows; penial hooks of small size (<0.04 mm in length), expanding at base, tip sharp and curved towards genital orifice (Fig. 5E); vagina (v) short, stout, about one third of total penis length; gametolytic duct (gd) a long tube not extending as far as albumin gland, gametolytic sac ovate (gs); free oviduct (fo) very short, oviduct (ov) enlarged and folded; prostate gland inconspicuous and bound to oviduct (Fig. 4A); talon (ta) small, very short and club shaped; hermaphroditic duct (hd) bearing long seminal vesicle (sv) about one and half times longer than the length from talon to branching point of seminal vesicle (Fig. 4C); vagina wall with a corrugated fold and pale brown vaginal hooks, about 8 hooks/200 µm^2^, hooks small (<0.03 mm in length) with pointed tip slightly curving away from genital orifice (Figs 5F, G).

**Animal:** Live specimens exhibit yellowish-red reticulated skin, and reddish tentacular retractor muscles are visible through the semi-transparent body (Fig. 2A).

##### Etymology.

The specific epithet “*dermapyrrhosa*” is derived from the Greek “*derma*” meaning “skin” and “*pyrrhos*” meaning “red or yellowish-red”.

##### Distribution.

This species is known only from the type locality, which is an isolated limestone hill reaching about 300 meters above mean sea level, and which is surrounded by the Korat Plateau.

##### Remarks.

Up to now, the only description of the reproductive system of a *Perrottetia* species was that of *Perrottetia gudei* from Vietnam in which the presence of streptaxid vaginal hooks were recorded for the first time, but without being figured (Schileyko 2000).

#### Perrottetia aquilonaris

Siriboon & Panha sp. n.

urn:lsid:zoobank.org:act:2E376204-2D0F-4021-B795-399E33C8A677

http://species-id.net/wiki/Perrottetia_aquilonaris

Figs 2B 3D–H 4D–F 5H–M 6B Table 1

##### Type material.

Holotype CUMZ 5003 (Fig. 3D). Measurement: height 3.9 mm, shell width 6.6 mm, and with 6 whorls. Paratypes NHMUK 20130064 (2 shells), SMF 341487 (1 shell), CUMZ 5004 (1 shell).

##### Other material examined.

Tam Phra Bumpenboon, Phan, Chiangrai: CUMZ 5005. Wat Tam Pha Jaruey, Pa-daet, Chiangrai: CUMZ 5006. Tam Maesuai, Maesuai, Chiangrai: CUMZ 5007. Tam Chiangdao, Chiangmai: CUMZ 5008. Pha Chu, Nanoi, Nan: CUMZ 5009. Tam Pha Nangkoi, Rongkwang, Phrae: CUMZ 5010.

##### Type locality:

Wat Tam Pha Plong, Chiangdao, Chiangmai, Thailand, 19°24'7.3"N, 98°55'5.6"E.

##### Diagnosis.

*Perrottetia aquilonaris* sp. n. can be distinguished from the similar south Indian species *Perrottetia watsoni* (Blanford, 1860) and *Perrottetia beddomii* (Blanford, 1899) by its smooth shell surface and the presence of a sinulus and a bifid columellar lamella. In comparison, *Perrottetia beddomii* possesses a supracolumellar lamella, while *Perrottetia watsoni* hasa second parietal lamella adjacent to the first parietal lamella (Blanford 1860, 1899, Kobelt 1906). *Perrottetia gudei* from North Vietnam differs from the new species in its larger, oblique-heliciform shell, which is less deviated from the vertical axis. A fine transverse ridge is present at the suture. A sinulus is absent, the peristome is much thicker, and bifid columellar lamella is absent (Fulton 1915). The genital system of *Perrottetia gudei* differs from that of this new species by possession of a long and slender penis, an entirely free long oviduct, a gametolytic duct and sac extending as far as the albumin gland, the absence of seminal vesicles and a more scattered arrangement of penial hooks (Schileyko 2000).

##### Description.

Shell suboblique-heliciform, white and translucent; teleoconch with 6 whorls, spire convex, suture indistinct; shell surface glossy, with transverse ridges diminishing below the periphery; embryonic shell large, about 2½ whorls, with smooth surface; following whorls regularly expanding; shell periphery shouldered, in apertural view left periphery of the penultimate whorl extending beyond the diameter of the last whorl; last whorl axially deflected; two deep and short longitudinal furrows present; umbilicus narrow (Fig. 3D); aperture subcircular, peristome discontinuous, thickened and expanded, short sinulus present, sometimes with a longer and tapering sinulus (Fig. 3H); apertural dentition consisting of one strong first parietal lamella, a small second parietal lamella separated at right angles, one small upper palatal lamella, one large palatal lamella, one basal lamella and a bifid columellar lamella (Fig. 3E).

**Figure 6.**
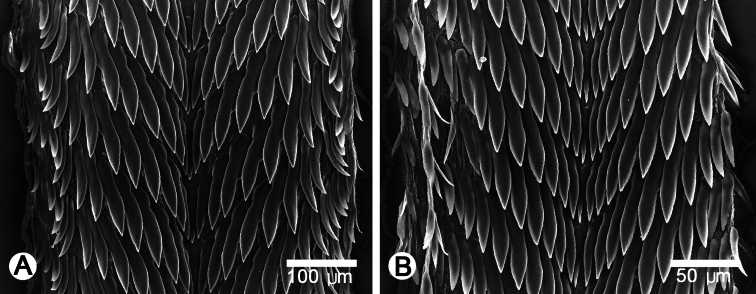
Radula morphology of **A** *Perrottetia dermapyrrhosa* sp. n. (paratype CUMZ 5002), and **B** *Perrottetia aquilonaria* sp. n. (paratype CUMZ 5004).

**Radula:** Teeth arranged in anteriorly V-shaped rows, each row containing 21–23 teeth with the formula (10-11)-1-(10-11); central tooth small, sharp, triangular with pointed cusp; lateral and marginal teeth undifferentiated, unicuspid and lanceolate; lateral teeth gradually reduced in length and size, outer teeth much smaller and shorter than inner teeth (Fig. 6B).

**Genital organs:** Atrium (at) short; penis tripartite, proximal part long and narrow, central section globular with a thick muscular wall, distal section again long and narrow; penial sheath (ps) thin, extends about half of total penis length; penial sheath retractor muscle (psr) very thin, originating at the atrium, inserting distally on penial sheath (Fig. 4D); vas deferens (vd) passes through about one-fifth of penial sheath length before entering into penis distally (Fig. 4E); penial retractor muscle (pr) thin and very long, inserting at penis and vas deferens junction; internal wall of atrium generally smooth with numerous pores (Fig. 5H); penial wall with scattered and pale brown penial hooks about 24 hooks/200 µm^2^; hooks located on papillae (pl), papillae separated by low reticulated folds; penial hooks of small size (< 0.02 mm in length), expanding at base, tip sharp and curved towards genital orifice (Figs 5J–L); vagina (v) short, stout, about a seventh of total penis length; gametolytic duct (gd) long but not extending as far as albumin gland; gametolytic sac ovate (gs); proximal free oviduct (fo) stout and distally enlarged; oviduct (ov) enlarged and folded; prostate gland inconspicuous and bound to oviduct (Fig. 4D); talon (ta) small, very short and club shaped; hermaphroditic duct (hd) bearing long seminal vesicle (sv) about one and half times longer than the length from talon to branching point of seminal vesicle (Fig. 4F); vaginal wall with parallel vaginal folds; vaginal hooks absent (Fig. 5M).

**Animal:** Live specimens exhibit yellowish reticulated skin, and pale yellowish tentacular retractor muscles are visible through the semi-transparent body (Fig. 2B).

##### Etymology.

The specific epithet is from the Latin “*aquilonaris*” meaning “north or northern”. It refers to the distribution range of this new species in northern Thailand.

##### Distribution.

This species is known from severallimestone areas in northern Thailand. The animals can be found in altitudes up to 200 meters above mean sea level.

##### Remarks.

Some variation has been observed in the sinulus and the bifid columellar lamella. Populations from Chiangmai and Chiangrai Provinces possess a longer and tapered sinulus (Figs 3G, H). Specimens collected between those two provinces have a shorter sinulus, and specimens from Chiangmai possess a large bifid columellar lamella (Fig. 3H).

#### Perrottetia phuphamanensis

Siriboon & Panha sp. n.

urn:lsid:zoobank.org:act:4E8CC516-99E8-4652-9B2A-1796689E2456

http://species-id.net/wiki/Perrottetia_phuphamanensis

Figs 3I–K Table 1

##### Type material.

Holotype CUMZ 5011 (Fig. 3I). Measurement: shell height 5.0 mm, shell width 6.9 mm, and with 6¼ whorls. Paratypes NHMUK 20130066 (2 shells), SMF 341488 (2 shells), CUMZ 5012 (14 shells).

##### Other material examined.

Tam Kangkao, Phuphaman, Khonkaen: CUMZ 5013.

##### Type locality.

Phuphaman National Park, Phuphaman, Khonkaen, Thailand, 16°45'34.0"N, 101°57'50.3"E.

##### Diagnosis.

*Perrottetia concinna* (Blanford, 1880) differs from *Perrottetia phuphamanensis* sp. n. in its smaller shell, higher spire and more distinct suture. The left periphery of the penultimate whorl does not extend beyond the diameter of the last whorl, the aperture is semi-ovate, and a bifid columellar lamella is present. *Perrottetia peroteti* differs from this new species in its fine transverse ridge at the suture, the smaller second parietal lamella, and the absence of the upper palatal and supracolumellar lamellae.

*Perrottetia gudei* can be distinguished from *Perrottetia phuphamanensis* sp. n. by its lower spire, a stronger transverse ridge at the suture, its triangular aperture, the second parietal lamella not being adjacent to the first parietal lamella and the absence of a supracolumellar lamella. Distinguishing features from *Perrottetia aquilonaria* sp. n.are the smaller shell and the presence of a sinulus. In addition, the left periphery of the penultimate whorl does not extend beyond the diameter of the last whorl, the peristome is thinner, the second parietal lamella is not adjacent to the first parietal lamella, and a bifid columellar and supracolumellar lamellae are both absent.

##### Description.

Shell suboblique-heliciform, white and translucent; whorls 6¼, spire convex, suture distinct; shell surface glossy, with a reduced transverse ridge; embryonic shell large consisting of about 2½ whorls, with smooth surface; following whorls regularly expanding; shell periphery shouldered, last whorl axially deflected; two shallow and short longitudinal furrows present; umbilicus narrow (Fig. 3I); aperture subcircular, peristome discontinuous, very thick and slightly expanded; apertural dentition with a large first parietal lamella and with a second parietal lamella adjoining at right angles; one upper palatal lamella, one palatal lamella, one basal lamella, one large strong columellar lamella, one small supracolumellar lamella (sometimes absent) (Fig. 3K).

##### Etymology.

The specific epithet is derived from the type locality of this new species, the Phuphaman National Park, Khonkaen Province.

##### Distribution.

This species is known only from the type locality.

##### Remark.

To date no living examples have been found.

**Table 1.** Shell measurements of the three new *Perrottetia* species. Specimen collections and catalogue numbers indicated in parentheses.

**Species and locality and CUMZ nos**	**No. of specimens**	**Ranges, mean ± S.D. in mm of:**				**Number of whorls**
		**Shell height**	**Shell width**	**H/W ratio**	**Shell angle**	
*Perrottetia dermapyrrhosa* sp. n.						
Wat Tam Namsrithong, Nong Kungsi, Kalasin: (5001, 5002)	7	5.4–6.6<br/> 6.2±0.39	7.4–8.1<br/> 7.7±0.26	0.7–0.9<br/> 0.8±0.06	14.2–28<br/> 21.1±5.01	6–6½
*Perrottetia aquilonaria* sp. n.						
Wat Tam Pha Plong, Chiangdao, Chiangmai: (5003, 5004)	5	3.7–4.3<br/> 4.0±0.23	6.3–6.6<br/> 6.4±0.13	0.6–0.7<br/> 0.6±0.04	19.8–38.0<br/> 27.5±7.59	6
Tam Phra Bumpenboon, Phan, Chiangrai: (5005)	5	4.0–4.3<br/> 4.2±0.12	6.9–7.4<br/> 7.2±0.20	0.6–0.6<br/> 0.6±0.01	23.3–34.9<br/> 28.7±4.14	5½–6
Wat Tam Pha Jaruey, Pa-daet, Chiangrai: (5006)	6	2.9–3.4<br/> 3.2±0.17	5.9–6.3<br/> 6.1±0.15	0.5–0.6<br/> 0.5±0.02	26.4–31.8<br/> 29.7±2.26	5½–6
Tam Maesuai, Maesuai, Chiangrai: (5007)	13	3.9–4.7<br/> 4.3±0.27	6.5–7.5<br/> 7.1±0.34	0.5–0.7<br/> 0.6±0.05	19.3–37.3<br/> 24.6±5.07	6
Km 93+200, Tam Chiangdao, Chiangmai: (5008)	11	3.6–4.1<br/> 3.7±0.14	5.7–6.4<br/> 6.1±0.26	0.6–0.7<br/> 0.6±0.03	21.5–29.4<br/> 24.9±2.48	5½–6
Pha Chu, Nanoi, Nan: (5009)	21	3.4–4.4<br/> 3.9±0.31	6.0–6.9<br/> 6.4±0.26	0.5–0.7<br/> 0.6±0.05	15.6–30.7<br/> 21.7±3.98	5½–6
Tam Pha Nangkoi, Rongkwang, Phrae: (5010)	25	2.9–3.9<br/> 3.3±0.25	5.5–6.1<br/> 5.7±0.17	0.5–0.7<br/> 0.6±0.04	17.6–35.8<br/> 25.7±3.91	5½–6
*Perrottetia phuphamanensis* sp. n.						
Phuphaman National Park, Khonkaen: (5011, 5012)	19	4.6–5.6<br/> 5.0±0.24	6.8–8.1<br/> 7.3±0.34	0.6–0.8<br/> 0.7±0.05	15.0–38.8<br/> 25.4±5.09	6–6½
Tam Kangkao, Phuphaman, Khonkaen: (5013)	11	4.9–5.5<br/> 5.1±0.20	6.9–7.7<br/> 7.2±0.28	0.7–0.8<br/> 0.7±0.04	16.8–31.1<br/> 22.6±4.20	6–6¼

## Discussion

The Streptaxidae were divided into 6 subfamilies and 3 new subfamilies by Schileyko (2000). Prior to Schileyko’s revision only two subfamilies, the Streptaxinae and the Enneinae had been recognized, which were primarily based on their shell morphology (Zilch 1960, Richardson 1988). Where material was available, Schileyko (2000) included characters from the genital organs to establish a revised system for the Streptaxidae. The Streptaxinae sensu Schileyko is composed of *Perrottetia* and 20 additional genera with discontinuous distribution patterns in South America, some Indian Ocean Islands, including the Mascarines and Seychelles (but excluding mainland Africa and Madagascar), South and Southeast Asia and the Philippines.

Records in the literature show *Perrottetia* having a tropical distribution in South Asia, Southeast Asia and some parts of East Asia. There is a concentration of 11 species in the Western and Eastern Ghats of peninsular India and two species are recorded from Sri Lanka, one of which is endemic (Naggs and Raheem 2000). Some South Asian species were discovered at high altitudes up to 4000 m above mean sea level. *Perrottetia* has also been recorded from Myanmar, Laos, North to South Vietnam and some parts of China such as Hainan Island and Taiwan, and is now recorded from Thailand.

Anatomical studies of streptaxids that included internal anatomy were pioneered by Stoliczka (1871) but, as pointed out by Sutcharit et al. (2010) and even following Schileyko’s (2000) revision, internal anatomy has been described for only 37 of the 60 currently recognized streptaxid genera, many of these in a very superficial way. In addition, in genera where reproductive anatomy has been investigated, only one or very few species may have been examined. Usually it is by chance that the reproductive anatomy of the type species of a genus could be investigated, which however is essential for the definition of the genus. Prior to this study, the reproductive anatomy of *Perrottetia* was only known for *Perrottetia gudei*. An important feature to note is that, unlike in other anatomically known members of the Streptaxinae, a part of the vas deferens passes under a section of the penial sheath (Schileyko 2000). There is clearly a long way to go before the system of the Streptaxidae can be considered as stable. For the future, research needs to focus on the accurate description of the morphological variation of both, shells and genital organs. These results should then be corroborated by molecular phylogenetic studies in order to better understand this fascinating group of land-snails.

## Supplementary Material

XML Treatment for Perrottetia

XML Treatment for Perrottetia dermapyrrhosa

XML Treatment for Perrottetia aquilonaris

XML Treatment for Perrottetia phuphamanensis
